# Right Atrial Myocardial Remodeling in Children With Atrial Septal Defect Involves Inflammation, Growth, Fibrosis, and Apoptosis

**DOI:** 10.3389/fped.2020.00040

**Published:** 2020-02-14

**Authors:** Hatem Rouatbi, Nesrine Farhat, Ruth Heying, Arlette Gérard, Jaime F. Vazquez-Jimenez, Marie-Christine Seghaye

**Affiliations:** ^1^Department of Pediatrics & Pediatric Cardiology, University Hospital Liège, Liège, Belgium; ^2^Department of Pediatric Cardiology, University Hospital Leuven, Leuven, Belgium; ^3^Department of Pediatrics, GIGA Neurosciences, University Hospital Liège, Liège, Belgium; ^4^Department of Pediatric Cardiac Surgery, University Hospital Aachen, Aachen, Germany

**Keywords:** myocardial remodeling, congenital heart disease, atrial septum defect, inflammation, growth, fibrosis, apoptosis

## Abstract

**Introduction:** Myocardial remodeling due to large atrial septum defect (ASD) is macroscopically characterized by dilation of the right-sided cardiac cavities secondary to volume overload, the cellular mechanisms of which are not yet understood. We postulated that inflammation, fibrosis, and cell death are actors of right atrial remodeling secondary to ASD.

**Patients and Methods:** In 12 children with large ASD (median age: 63 months), expression of genes coding for proteins involved in the response to cell stress and -protection, inflammation, growth and angiogenesis, fibrosis, and apoptosis was assessed by RT-PCR in right atrial myocardial biopsies taken during cardiac surgery. The presence of cytokines in myocardial cells was confirmed by immunohistochemistry and effective apoptosis by TUNEL assay.

**Results:** In all patients investigated, a cellular response to early mechanical stress with the initiation of early protective mechanisms, of inflammation (and its control), -growth, and -angiogenesis, of fibrosis and apoptosis was present. The apoptotic index assessed by TUNEL assay averaged 0.3%.

**Conclusions:** In children with large ASD, macroscopic right atrial remodeling relates to cellular mechanisms involving the expression of numerous genes that either still act to protect cells and tissues but that also harm as they initiate and/or sustain inflammation, fibrosis, and cell death by apoptosis. This may contribute to long term morbidity in patients with ASD.

## Introduction

Atrial septal defect (ASD) is a common congenital heart disease responsible for inter-atrial left-to-right shunt and for volume overload of the right cardiac cavities and their dilation. Hemodynamic overload initiates myocardial remodeling that comprises changes in tissue properties secondary to the activation of different signal cascades such as inflammatory-, growth- and death signaling pathways. Consecutive cardiomyocyte loss due to cell death or phenotype transformation in cardio-myofibroblasts ends up in myocardial fibrosis and finally in systolic and/or diastolic myocardial dysfunction. In patients with ASD, right atrial remodeling may elicit late supra-ventricular arrhythmias.

Mechanisms of myocardial remodeling are complex and have principally been studied in models of ischemic myocardial injury or pressure overload, in particular in systemic hypertension (1). In infants, mechanical stress related to pressure overload of the right ventricle leads to myocardial expression of pro-inflammatory cytokines mainly via the activation of p38MAPK signaling (2). Nevertheless, little information is available about the effect of volume overload on the pathophysiology of myocardial remodeling.

This study was therefore designed to investigate mRNA expression of genes coding for factors involved in mechanical stress (ANF), cell protection [c-Fos, Heat Shock Protein (HSP)-70, HSP-90], inflammation [Tumor necrosis factor (TNF)-α, Interleukin (IL)-1β, IL-6, IL-10], growth and angiogenesis [Cardiotrophin (CT)-1, Hypoxia Inducing Factor (HIF)-1α, Vascular Endothelial Growth Factor (VEGF), Insulin-Like Growth Factor (IGF)-1], fibrosis of the extracellular matrix [Tissue Growth Factor (TGF)-β, the amino-terminal peptide of Type III procollagen PIIIP, Collagen III], and regulation of apoptosis (Fas Ligand, Bak, Bcl-xL) in children with volume overload of the right atrium due to atrial septal defect.

## Patients and Methods

According to the current recommended strategy, the large majority of patients undergo nowadays percutaneous- instead of surgical ASD closure. Because only few patients are still scheduled for surgical closure we recruited those matching the criteria for entering the study from our two institutions where the myocardial samples were processed and analyses performed. For that reason, data of both patient groups were not pooled but presented separately.

Inclusion criteria were (1) pre-pubertal state in children aged <10 years of age; (2) the presence of an isolated ASD with hemodynamic relevant left-to-right shunt requiring closure. Exclusion criteria were (1) syndromic affection; (2) genetic disorder; (3) infectious or inflammatory state.

The ethics committees of the University Hospital Aachen (Institution I) and the University Hospital Leuven (Institution II) gave their agreement, respectively. Parents gave their informed and written consent. A total of 12 children were enrolled, divided into 2 groups according to the institution of origin. Group 1, *N* = 7 (females, *n* = 5), Institution I, median age: 78.8 months; Group 2, *n* = 5 (females, *n* = 5), Institution II, median age: 49.6 months.

All patients had an ASD type *ostium secundum* but one who had an ASD type superior *sinus venosus*. All patients showed significant left-to-right shunt as shown by clinical examination (hyperactivity of the right ventricle, systolic murmur of a relative pulmonary stenosis with or without diastolic tricuspid rumble), electrocardiography (right ventricular hypertrophy) and echocardiography (enlarged right atrium, -right ventricle, and -pulmonary artery). Left-to right shunt was calculated by applying the Fick formula during heart catheterization that was performed under general anesthesia at least 4 weeks before surgery in 5 patients of group I who were initially scheduled for interventional ASD closure.

### Sampling of Myocardial Biopsies

In all cases and in both institutions, conventional general anesthesia consisted of midazolam, fentanyl sulfate, and pancuronium bromide. A biopsy (3 mm^3^) was taken from the right atrial appendage immediately before institution of cardiopulmonary bypass, during right atriotomy.

Biopsies were immediately snap-frozen in liquid nitrogen and stored at −80°C until processed.

### Quantitative Real Time Reverse Transcriptase-Polymerase Chain Reaction (RT-PCR)

In group I, RNA isolation was performed with RNeasy® Micro-Kit (QIAGEN, Hilden, Germany), according to the manufacturer's recommendation.

Isolated RNA was reverse transcribed to complementary DNA (cDNA) using iScriptTM cDNA Synthesis Kit (BioRad, Germany). A standard for each primer set was generated for quantitative real-time reverse transcription-polymerase chain reaction (qRT-PCR) by cloning PCR products in pBluescript and the identity was verified by sequencing. A 2 μl cDNA sample was incubated with 20 μl QuantiTect Mix containing fluorescence dye SYBR Green (QIAGEN Hilden® Germany) and 0.6 μmol/l of each primer pair. PCR amplification was performed after initial denaturation at optimized annealing temperatures for each primer pair using MJ Research Opticon 2 (Biozym). Melting curves were acquired by stepwise increase of the temperature from 55° to 95°C. Threshold cycles (CTs) of real-time PCR curves were determined by Opticon® Monitor software (Biorad, Hercules, CA). The difference of the CTs (ΔCT) of targets and 18S-RNA housekeeping control gene reflected the amount of target mRNA in each sample. Target mRNA was quantified according to standard curve and normalized to levels of 18S-RNA.

In Group II RNA was also isolated by using the RNeasy kit according to the manufacturers recommendations (QIAGEN Inc., Hilden, Germany). Total mRNA concentration was assessed by photometric analyses by measuring absorbance at 260 nm, using a NanoDrop 2000-C apparatus (Thermo Scientific, Wilmington, DE, USA).

RNA (2 μg) was reverse-transcribed to complementary deoxyribonucleic acid (DNA) with random hexamers. Two microlitre of cDNA samples were incubated with 20 μl of Quanti Tect Mix containing fluorescing SYBR Green (Qiagen GmbH, Hilden, Germany) and in each case 0.6 μl of the primer pairs. The real-time cycler conditions consisted of an initial activation PCR step of 15 min at 95°C, and then 40 cycles of 15 s at 94°C, 30 s at 55°C, and 30 s at 72°C. Three replicas were performed for each analyzed sample, and positive and negative controls were tested on each plate.

SYBR Green served as the DNA dye to quantify the PCR products. Expression was normalized to levels of actin-mRNA and calculated with 2^−ΔCT^. Primers used in both groups are listed in Table 1.

**Table 1:** RT-PCR primer pair sequences in group I and in group II.

**Genes**	**RT-PCR Primer pair sequence**	
	**Group 1**	**Group 2**
ANF	AGTTCAGAGGATGGGCACAC ATCACAACTCCATGGCAACA	
c-Fos	TTTATAGTGGGCGGAAGTGG ACGTCCTGGACAAAGGTCAC	
HSP-70	CCGAGAAGGACGAGTTTGAG AATCTTGGAAAGGCCCCTAA	AACTACAAGGGCGAGAACCGGTC GATGATCCGCAGCACGTTCAGA
HSP-90	CGCATGAAGGAGACACAGAA TCCCATCAAATTCCTTGAGC	TGCGGTCACTTAGCCAAGATG GAAAGGCGAACGTCTCAACCT
TNF-α		GGAGCCAGCTCCCTCTATTT GGCTACATGGGAACAGCCTC
IL-1β		CTGTCCTGCGTGTTGAAAGA TTCTGCTTGAGAGGTGCTGA
IL-6		AACCTGAACCTTCCAAAGATGG TCTGGCTTGTTCCTCACTACT
IL-10		CTGTCCTGCGTGTTGAAAGA TTCTGCTTGAGAGGTGCTGA
CT-1	AACTCTTGGACCCTCCTCGT TAAGGAAGCCAGCCAAGAGA	CACTTGGAGGCCAAGATCC TCTCCCTGGAGCTGCACAT
HIF-1α	TGATGACCAGCAACTTGAGG TTGATTGAGTGCAGGGTCAG	GCACAGGCCACATTCACGTATAT GGTTCACAAATCAGCACCAAGC
VEGF	CCCACTGAGGAGTCCAACAT TTTCTTGCGCTTTCGTTTTT	CTGTCTAATGCCCTGGAGCC ACGCGAGTCTGTGTTTTTGC
IGF-1		CAGCCCCCATCTACCAACAA GCACTCCCTCTACTTGCGTT
TGF-β		CACCATCGAGAGTTCCGGTT AAGCGTTCCCGGATGTAGTC
PIIIP	TAAACAACTGGGTGCCTTCC CAGCAAGTCCTTCCCAAGAG	
Collagen III		CCTTCGACTTCTCTCCAGCC TTTCGTGCAACCATCCTCCA
Fas-L	GATGGAGGGGAAGATGATGA TGGAAAGAATCCCAAAGTGC	
Bak	GGGTCTATGTTCCCCAGGAT GCAGGGGTAGAGTTGAGCAG	GAGGATCTACAGGGGACAAGT CTGAGTGGGAGCCCAGTTTC
Bcl-xL	GGCTGGGATACTTTTGTGGA GGGAGGGTAGAGTGGATGGT	GGTGAATGGAGCCACTGCG CTTTACTGCTGCCATGGGGA
18sRNA	AAACGGCTACCACATCCAAG CCTCCAATGGATCCTCGTTA	
Actin		AGAGCTACGAGCTGCCTGAC AGCACTGTGTTGGCGTACAG

### Immunohistochemistry and TUNEL Assay

In group II, frozen sections of 4 μm were mounted on polylysine-coated glass slides. Sections were rehydrated through graded ethanol (100% for 5 min, ×2; 95% for 5 min, 80% for 3 min; 70% for 3 min; ddH_2_O for 5 min). Epitope unmasking was achieved by incubating the sections in a humidified chamber with proteinase K (20 μg/ml; Sigma) for 15 min at room temperature. Endogenous peroxidase activity was then blocked by incubating the sections in a humidified chamber for 15 min at 23°C in 3% H_2_O_2_ and incubated for 40 min at 23°C with blocking solution (PBS + 10% serum). The sections were then incubated at 23°C for 1 h with either anti-CT-1 (MAB2602. R.D Systems) (5 μg/ml) and or anti-IL1β (ab8320. Abcam) washed in PBS (PBS, Tween 20; 5 min. ×3), and incubated further at 23°C for 1 h with secondary antibody (VC001-050 R.D Systems and ab150113, Abcam respectively). After washing in PBS (5 min. x 3), the sections were counterstained with hematoxylin for 2 min and DAPI for 1 min respectively. After dehydration through graded ethanol solutions and xylenes, glass coverslips were mounted on slides in Permount (Sigma).

TUNEL assay was performed with the *in situ* Cell Death Detection Kit, TMR red (TUNEL; ≠ 12156792910 Roche Applied Science) according to manufacturer's instructions.

### Statistical Analysis

Results are expressed as median value and interquartile range or as mean value ± SEM. Correlation of independent parameters was assessed by the Pearson correlation test. A *p* < 0.05 was considered significant. Data were analyzed with statistical Package for Social Science (IBM SPSS Software 20.0).

## Results

Demographic and clinical patient data are summarized in Table 2. Results of mRNA expression measured in both groups were not pooled but analyzed separately. Table 3 summarizes mRNA concentrations of the genes of interest measured in group I and/or in group II. In all patients tested, expression of mRNA coding for following genes was detected: ANF as a marker of mechanical stress, c-Fos, HSP-70, HSP-90, the immediate-early genes providing protective mechanisms, the pro-inflammatory TNF-α, IL-1β, IL-6, the growth- and angiogenesis controlling factors CT-1, HIF-1α,VEGF, IGF-1, of those regulating apoptosis (pro-apoptotic Fas-L and Bak, anti-apoptotic Bcl-xL) or fibrosis of the extracellular matrix (TGF-β, PIIIP, Collagen III). mRNA coding for the anti-inflammatory IL-10 was detected in 5 out of the 7 patients of group II. In all patients, the expression of Bcl-xL-mRNA was higher than that of Fas-L-mRNA or Bak-mRNA. Expression of any of the target mRNAs tested was not correlated with patient age.

**Table 2:** Demographic and clinical patient data in group I and in group II.

	**Group 1**	**Group 2**
	***n*** **=** **7**	***n*** **=** **5**
Age (months)	78^*^ (6)	53^*^ (6.5)
Gender		
Female (*n*)	5	5
Male (*n*)	2	0
ASD *ostium secundum* (*n*)	6	5
ASD *sinus venosus* (*n*)	1	0
Left-to-right shunt (%)	63 (15.4)^**^	–
Right atrial pressure (mmHg)	7 (4.75)	–

Results are shown as number or as median value (interquartile range).

ASD, atrium septum defect.

* P < 0.05 between both groups.

** *n = 5*.

**Table 3:** Intra-myocardial concentrations of mRNA coding for target genes in group I and group II.

**Gene-mRNA**	**Group 1**	**Group 2**
	***N*** **=** **7**	***N*** **=** **5**
ANF	89.646 ± 2.92	
c-Fos	51.934 ± 2.50	
HSP-70	86.65 ± 2.68	0.36 ± 0.74^*^
HSP-90	105.54 ± 2.51	4.20 ± 3.56^*^
TNF-α		0.43 ± 0.08^*^
IL-1β		0.69 ± 0.83
IL-6		3.22 ±1.79^*^
IL-10		1.19 ± 0.16
CT-1	61.98 ± 2.00	0.54 ± 0.18^*^
HIF-1α	77.97 ± 3.09	
VEGF	57.629 ± 2.28	3.24 ± 2.58^*^
IGF-1		0.70 ± 0.48^*^
TGF-β		10.8 ± 0.22
Fas-L	37.094 ± 1.67	
Bak	49.57 ± 1.80	0.81 ± 0.29^*^
Bcl-xL	72.13 ± 2.03	3.98 ± 193
PIIIP	67.98 ± 1.98	
Collagen III		0.95 ± 0.41^*^

Concentrations are normalized for 18s in group I and for actin in group II.

* *Indicates n = 4*.

In patients in whom cardiac catheterization was performed before surgery, the amount of left-to-right shunt and mean right atrial pressure were not correlated with the mRNA-expression of genes of interest, respectively.

**Figure 1:**
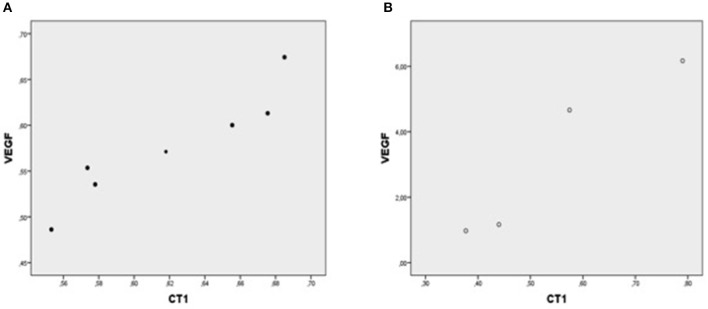
Plot chart showing the positive correlation between myocardial expression of CT-1-mRNA and VEGF-mRNA in group I (*n* = 7) **(A)** and in group II (*n* = 4) **(B)**. Pearson correlation coefficient co-efficient: 0.94, *p* = 0.002 in group I and 0.95, *p* = 0.04 in group II, respectively.

**Figure 2:**
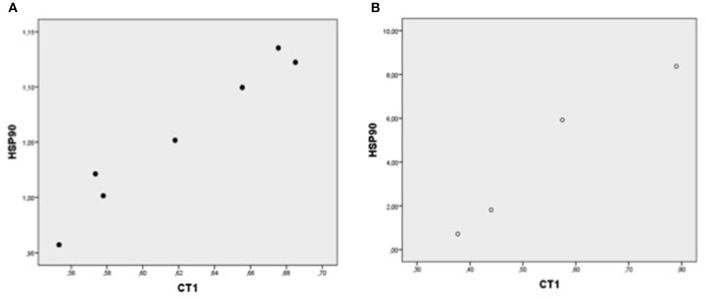
Plot chart showing the positive correlation between myocardial expression of CT-1-mRNA and HSP90-mRNA in group I (*n* = 7) **(A)** and in group II (*n* = 4) **(B)**. Pearson correlation coefficient: in group: *p* = 0.000, and group II, *p* = 0.024, respectively.

**Figure 3:**
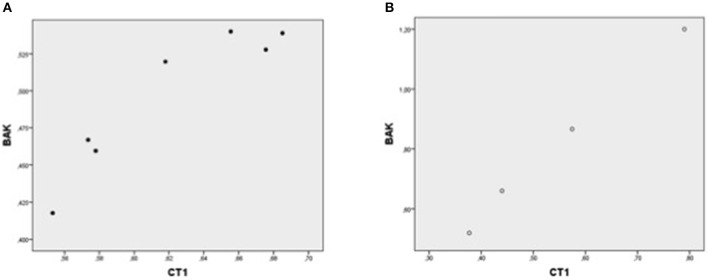
Plot chart showing the positive correlation between myocardial expression of CT-1-mRNA and BAK-mRNA in group 1 (*n* = 7) **(A)** and in group 2 (*n* = 4) **(B)**. Pearson correlation coefficient: 0.93 in group 1, *p* = 0,002 and 0.99 in group 2, *p* = 0.002, respectively.

**Figure 4:**
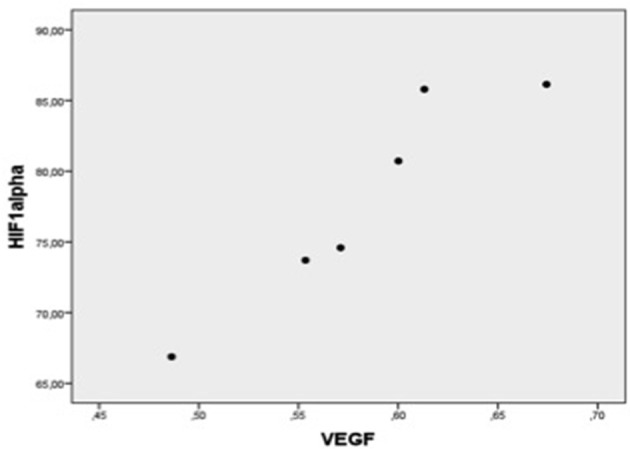
Plot chart showing the positive correlation between myocardial expression of VEGF-mRNA and HIF-1α-mRNA in patients of group 1 (*n* = 6). Pearson correlation coefficient = 0.95, *p* = 0.004.

**Figure 5:**
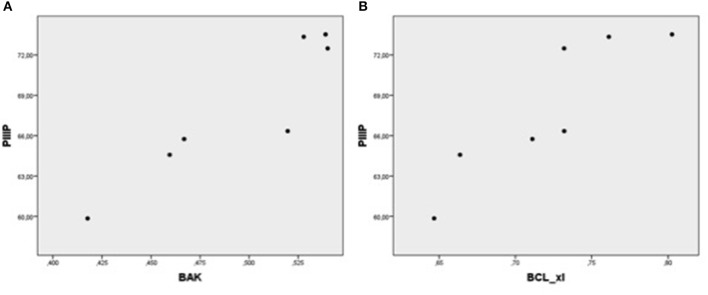
Plot chart showing the positive relationship between the expression of PIIIP-mRNA and BAK-mRNA **(A)** and between PIIIP-mRNA and Bcl-xL-mRNA **(B)** in patients of group 1 (*n* = 7). Pearson correlation coefficient: 0.93, *p* = 0,003 and 0.90, *p* = 0.006, respectively.

In each patient group, expression of mRNA coding for genes implicated in cell protection, growth, angiogenesis, apoptosis but inflammation generally correlated with each other (Table 4, Figures 1–5).

**Table 4:** Exemplary correlations between expression of mRNA coding for protective proteins, growth factors, and regulators of apoptosis that were present in both groups.

	**CT-1**		**VEGF**		**HSP70**		**HSP90**		**BAK**	
	**Group I**	**Group II**	**Group I**	**Group II**	**Group I**	**Group II**	**Group I**	**Group II**	**Group I**	**Group II**
CT-1	–	–	*R* = 0.94	*R* = 0.95			*R* = 0.98	*R* = 0.97	*R* = 0.93	*R* = 0.99
			*p* = 0.002	*p* = 0.04			*p* = 0.000	*p* = 0.024	*p* = 0.002	*p* = 0.002
VEGF	*R* = 0,94	*R* = 0,95	–	–	–	–	*R* = 0.93	*R* = 0.99	*R* = 0.90	*R* = 0.96
	*p* = 0.002	*p* = 0,04					*p* = 0.002	*p* = 0.006	*p* = 0.006	*p* = 0.04
HSP70	–	–	–	–	–	–	*R* = 0.794	*R* = 0,963	–	*R* = 0.952
	–						*p* = 0.033	*p* = 0.037		*p* = 0.048
HSP90	*R* = 0.98	*R* = 0.97	*R* = 0.93	R= 0,99	R=0,79	*R* = 0.96	–	–	*R* = 0,94	*R* = 0.98
	*P* = 0.000	*p* = 0.024	*p* = 0.002	*p* = 0.006	*p* = 0,033	*p* = 0.037			*p* = 0.002	*p* = 0.023
BAK	*R* = 0.93	R= 0.99	*R* = 0.90	*R* = 0.96	–	*R* = 0.95	*R* = 0.94	*R* = 0.98	–	–
	*p* = 0.002	*p* = 0.002	*p* = 0.006	*p* = 0/04		*p* = 0.048	*p* = 0.002	*p* = 0.023		

R, Pearson correlation coefficient.

*P < 0.05 is considered significant*.

In all patients tested of group II, mRNA expression of TGFβ negatively correlated with that of Bcl-xL (Pearson correlation coefficient: −0.92, *p* = 0.029).

### Immunohistochemistry and TUNEL Assay

**Figure 6:**
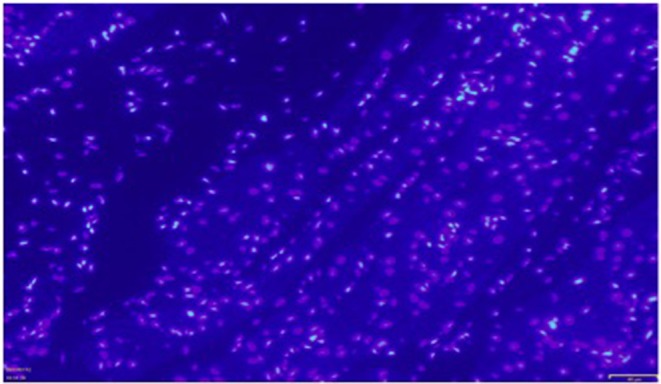
Exemplary immunocytochemistry study showing the presence of IL1β (green fluorescent staining) in cardiomyocytes of the right atrial myocardium. Magnification: x 200. The scale in the lower right corner represents 40 μm.

**Figure 7:**
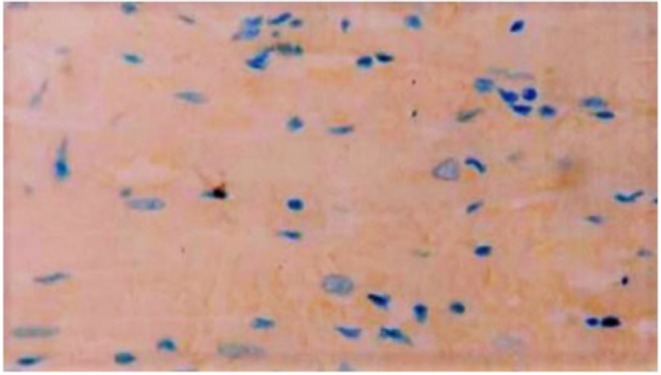
Exemplary immunocytochemistry study showing the presence of CT-1 (blue staining) in cardiomyocytes of the right atrial myocardium. Magnification: x 400.

Immunohistochemistry showed the presence of IL 1β and CT-1 in right atrial cardiomyocytes in all patients tested (Figures 6, 7).

**Figure 8:**
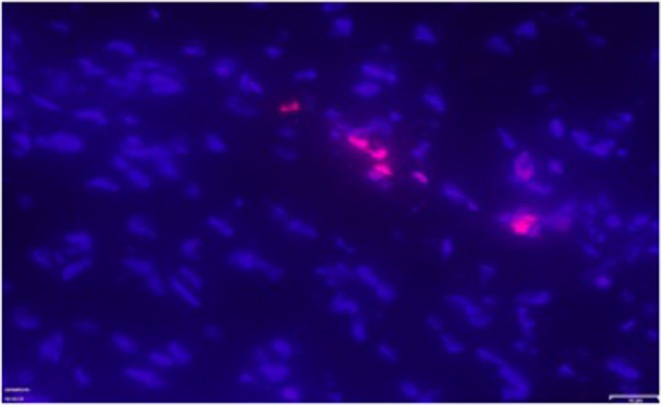
Exemplary positive TUNEL staining (pink fluorescent staining) in the right atrial myocardium of one patient with ASD. Magnification: x 400.

TUNEL positive cardiomyocytes were clearly detected in all patients with ASD (Figure 8). Apoptotic index, that is, the number of TUNEL-positive cardiomyocytes was 0.3%.

## Discussion

In this series, we assessed mRNA-expression of genes implicated in myocardial remodeling in the volume overloaded RA of children with large ASD.

In all patients tested, the mRNA expression pattern indicated a cellular response to mechanical stress, the initiation of early protective mechanisms, of inflammation, growth, and angiogenesis, of fibrosis and apoptosis. The presence of the non-constitutive inflammatory cytokine IL-1β and of the major growth factor CT-1 in the cardiomyocytes was confirmed at protein level by immunohistochemistry and the execution of apoptosis by the detection of DNA degradation products, respectively.

The expression of ANF-mRNA in our patients reflects the immediate biological response to mechanical stress of the volume overloaded RA-myocardium secondary to the large inter-atrial left-to right shunt. Accordingly, several studies have reported increased intra-myocardial and also circulating levels of ANF in patients with ASD (3). ANF possesses well-known biological effects beneficial to the overloaded cardiac cavities and may prevent pathological remodeling (4). Our study shows that other protective mechanisms are also initiated in the volume overloaded RA-myocardium. Indeed, mRNA expression of early markers of cellular stress such as c-fos, HSP-70 and HSP-90, that are well-known to play cyto-protective roles in numerous situations of injury (5, 6). c-Fos is a proto-oncogen that is part of Activator Protein-1, a transcription factor complex involved in cell survival and cell differentiation (5). It takes also part in the complex interplay underlying extra-cellular matrix (ECM) regulation by TGF-β and seems to be essential for the induction of the tissue inhibitor of metalloproteinases (Timp)-1 gene expression (7). Beside this, HSP70 and HSP-90 are molecular chaperones that play an important role in maintaining adequate protein folding in stressed cells and prevent apoptosis (6). HSP90 has been recognized to be involved in adaptive ventricular myocardial remodeling by modulating Angiotensin II-mediated hypertrophy via the nuclear factor (NF)kB pathway (8). In our patients, expression of HSP90-mRNA was correlated with that of mRNA coding for genes implicated in cell growth, angiogenesis, apoptosis and fibrosis, suggesting that chaperone proteins might be involved in RA-myocardial remodeling secondary to large ASD. In our study, all patients tested showed right atrial expression of mRNA coding for TGFβ, the major promotor of tissue fibrosis (9) the levels of which increase in the pressure-overloaded myocardium during hypertrophic growth (10). Furthermore, they showed also expression of mRNA coding for PIIIP, a marker of collagen-3 production, the serum levels of which are elevated in children with congenital cardiac disease (11), reflecting the activation of fibrosis-inducing pathways in the RA myocardium of our patients with ASD. Inflammatory cytokines are well-recognized to play a central role in inducing and controlling fibrogenesis (12) and participate in the structural changes that are characteristic for adaptive remodeling such as cardiomyocyte hypertrophy and for maladaptive events such as cardiac dilatation and heart failure (13). We detected mRNA coding for the pro-inflammatory cytokines TNF-α, IL-1β, and IL-6 and confirmed the presence of IL-1β at protein level in the RA myocardium of our patients. These cytokines are not constitutively expressed in the normal heart (14). This is in line with our previous observations showing that hemodynamic overload leads to myocardial up-regulation of pro-inflammatory cytokines by activating the NFkB and p38-MAPK pathways (2). This latter plays also a crucial role in cell differentiation, growth, and apoptosis (15). Besides its cardio-depressive effect TNF-α produces structural changes in the myocardium including cardiomyocyte hypertrophy, ventricular dilatation, rapid collagen decrease and loss of interstitial collagen network organization (16). TNF-α produced by stressed or injured cardiomyocytes initiates inflammatory phenotype transformation of cardiac fibroblasts that in turn express IL-1β and IL-6 (17, 18). IL-1β is an early pro-inflammatory cytokine also up-regulated in response to mechanical stress. It participates to adaptive cardiomyocyte hypertrophy by inducing IGF-1 (19). IL-1β promotes a matrix-degrading phenotype in cardiac fibroblasts with up-regulation of the synthesis of matrix metalloproteinases and plays an important role in extracellular matrix organization in response to mechanical or ischemic stress (20–22). Beside the expression of pro-inflammatory genes, the majority of our patients tested showed expression of IL-10 at mRNA level, suggesting the presence of a myocardial anti-inflammatory response in this situation. IL-10 belongs to the IL-10 cytokine family. It suppresses TNF-α and IL-1β synthesis by activating one of its target genes, the Suppressor of Cytokine Signaling (SOCS)-3, thus preventing from collagen synthesis by activated myofibroblasts (23) and from myocardial fibrosis (24).

IL-6 is the major regulator of the systemic inflammatory response that plays a protective role in the acute injured myocardium by preventing apoptosis, while it depresses contractility in chronically exposed myocardium (25). In a mouse model of chronic β-adrenergic stimulation, its secretion by activated fibroblasts leads to myocyte stimulation and finally to myocardial hypertrophy (26). In our series, myocardial mRNA-IL-6 expression suggests a stimulating effect of TNF-α and IL-1β on it and the role of IL-6 in the remodeling process (25, 27).

CT-1 is another member of the IL-6 family of cytokines and the most potent growth factor involved in myocardial hypertrophy (28). In recent studies, a role of CT-1 in extracellular matrix organization has been suggested (29). After myocardial infarction, CT-1 expressed in infarcted tissue has paracrine effects on neighboring fibroblasts of the adjacent viable myocardium. As a consequence, fibroblasts proliferate, and migrate to restore cellularity of the injured myocardium (30). This proliferative, migratory phenotype counterbalances the myofibroblast phenotype induced by TGF-β and Angiotensin II (31, 32). However, CT-1 may also mediate myocardial dysfunction as it has been suggested in our previous study where myocardial CT-1-expression in patients with cyanotic cardiac defect correlated with Troponin-I degradation (32). Besides its autocrine and paracrine effects, CT-1 increases the synthesis of HSP, in particular HSP 90 via the CT-1/ p42/44MAPK/NFkb-IL-6 signaling pathway (33). Our results showing a significant relationship between CT-1-mRNA and HSP90-mRNA in all patients of both groups suggests that this pathway might be implicated in the event cascade of RA myocardial remodeling in patients with ASD.

The presence of IGF-1-mRNA in the RA myocardium of our patients indicates a possible protective role against fibrogenesis as it has been shown in an animal model of partial IGF-1 deficiency (34).

Mechanical stretch due to increased wall expansion activates several pathways as discussed above, among others stretch-activated channels and the phosphatidylinositol 3-pathway. These latter are responsible for the increase of the HIF-1α subunit of HIF-1 that initiates in turn the expression of its target gene VEGF via the activation of the ERK /c-Fos signaling pathway (35). The presence of HIF-1α-mRNA and VEGF-mRNA in the RA of our patients suggests an implication of these pathways in the biological response to volume overload of the RA. The correlations we found between levels of VEGF-mRNA and levels of mRNA coding for HSP90, CT-1 and Bak, respectively, reflect the complex interplay between protective- and growth pathways and programed cell death in this clinical model of volume overload.

Myocardial apoptosis has been documented in response to a variety of cardiac stresses, including ischemia-reperfusion injury, and heart failure (36–38). In our series, genes regulating apoptosis were expressed at mRNA level in the RA myocardium and apoptotic bodies were detected at TUNEL staining. This is concordant with a previous study demonstrating increased apoptosis in the RA of patients with ASD, most of them being adults (39). However, in our patients, concentrations of Bcl-xL-mRNA were higher than those of Bak and FasL and negatively correlated with those of TGF-β-mRNA, suggesting that anti-apoptotic signals might predominate over pro-apoptotic ones in preschool children with ASD and prevent fibrosis. In this series, we were not able to provide any correlation between indicators of right atrial stretch such as the amount of left-to-right shunt or right atrial pressure measured during cardiac catheterization and the expression of mRNA coding for genes involved in myocardial remodeling. Given the complexity of the interactions between the different signaling pathways involved (12, 23), such a linear correlation is hardly to be expected in human tissue and must be tested in an experimental setting.

Cardiac surgery itself is a source of inflammatory signaling as we have demonstrated previously (40). However, given that the myocardial samples were taken before atrial cannulation and initiation of cardio-pulmonary bypass, we assume that the systemic inflammatory response that is not yet measurable by an elevation of circulating levels of pro-inflammatory cytokines at this stage (41) did not impact the intra-myocardial expression of the genes of interest in the present work.

### Limitation Section

The small number of patients included in this descriptive study is a limitation factor as is the absence of correlation between the expression pattern of mRNA coding for genes involved in myocardial remodeling and clinical outcome variables. The rarity of surgeries for ASD closure motivated us to include patients the myocardial tissue of whom was processed in 2 different institutions. Their results were therefore not pooled. The fact that similar results were obtained in both patient groups may however be considered strength of the study.

## Conclusion

Our results show that in children with ASD, myocardial remodeling of the RA involves inflammatory-, growth- fibrogenic- and apoptotic signaling pathways that are likely to be activated in response to cellular stress. Besides the expression of harmful mediators such as pro-inflammatory cytokines, protective mediators are also expressed that may delay the apparition of irreversible tissue remodeling. With this respect, the predominance of anti-apoptotic over pro-apoptotic regulators in children could be understood as an intrinsic myocardial protection allowing lesion reversibility after ASD-closure.

## Data Availability Statement

The raw data supporting the conclusions of this article will be made available by the authors, without undue reservation, to any qualified researcher.

## Ethics Statement

The studies involving human participants were reviewed and approved by Ethical Committee of the University Hospitals Leuven and Aachen. Written informed consent to participate in this study was provided by the participants' legal guardian/next of kin.

## Author Contributions

HR and M-CS conceived and designed the study. HR, AG, NF, RH, and JV-J contributed to the data acquisition, data analysis, and interpretation. HR and M-CS drafted the manuscript. All authors revised the manuscript critically and approved it finally before publication and agree to be accountable for all aspects of the work in ensuring that questions related to the accuracy or integrity of any part of the work are appropriately investigated and resolved.

### Conflict of Interest

The authors declare that the research was conducted in the absence of any commercial or financial relationships that could be construed as a potential conflict of interest.
